# Prolapse and Procidentia

**Published:** 1910-08-13

**Authors:** Aslett Baldwin


### Hospital Clinics.

### PROLAPSE AND PROCIDENTIA.

(A Lecture delivered at St. Mark's Hospital, City Road, on Friday, July 8, 1910.)

By ASLETT BALDWIN, F.R.C.S.

Gentlemen,—Many definitions have been given as to what is prolapse and what is procidentia. They both mean the same thing. I shall call prolapse that condition in which only the mucous membrane comes down, and procidentia that condition in which all the coats of the bowel come down.

The causes of this falling of the bowel are: (1) anything which causes increased straining and peristalsis, such as diarrhœa, worms, foreign bodies in the bowel, hard motions, ulcers in the bowel, proctitis, stricture of the urethra, phimosis, stone in the bladder or in the urethra, cystitis, an enlarged prostate. Also (2) anything which causes a generally relaxed condition of the patient's tissues, such as exhausting diseases, anything which interferes with the nervous power of the anus, over-stretching of the sphincter, or damage to the sphincter during operations. Another (3) cause is anything which produces a constant dragging on the mucous membrane of the bowel, such as polypi, tumours, and stricture of the bowel, anything which causes effusion between the mucous membrane of the bowel and its outer coats, and any inflammatory œdema.

### Varieties of Prolapse.

Prolapse of the mucous membrane may be either partial or complete; that is, part of the circumference may come down, or the whole ring of mucous membrane may do so. In the former case the prolapsed portion is in longitudinal folds. When the whole of the bowel comes down the folds are circularly arranged round the bowel. It may take place in different ways. The prolapse may start at the skin edge of the anus, or higher up than that, so that there will be a sulcus between the bowel and the anal canal. Perhaps you may be able to pass your little finger up, or possibly a probe. Or it may be that the bowel higher up comes down and does not show through the anus at all. These forms will be distinguished from an intussusception by the fact that there is no intestinal obstruction, no passage of blood, nor any of the other symptoms of intussusception.

### Treatment.

Now as to the treatment of these conditions. First try to eradicate any of the causes I have mentioned. Take the condition in children. By far the larger number of children can be cured by purely conservative treatment. By that I mean that the patient should be kept in bed, and whenever the bowels act the patient should be forced to lie on his side, and the uppermost buttock dragged slightly upwards by the nurse, so as to make the anal orifice somewhat oblique. If the patient has any straining when the bowels act, it is well to give an enema of cold water previously, and not to let him start the act until he is obviously quite ready. If, with those precautions, the bowel still comes down, the buttocks will have to be strapped together by strapping. And it is important to use a strapping which does not irritate the skin; it should be free from the resinous substances which are often incorporated. This strapping should be put from one great trochanter right round to the other. The whole of the anus should not be covered in, so that it will not be necessary, every time the bowel is opened, to remove part of the strapping; otherwise there may be ulceration of the skin. In addition, the child will require feeding up, and tonic treatment—cod-liver oil, iron, hypophosphites, arsenic, etc. In that way by far the larger number of cases of prolapse in children will be cured. But as long as there is any tendency to prolapse, the child should be kept lying on its back, and always made to defæcate in the left lateral position. That is much less likely to allow prolapse than if the patient defæcates while lying on the back. Similar treatment will also often cure the condition of prolapse of mucous membrane in youngish adults. In old debilitated people it probably will not be sufficient.

### Operative Treatment.

There are a number of operations which can be done, varying with the severity of the condition. Many cases of slight prolapse of mucous membrane are cured in the course of operations for piles. A very excellent way of treating prolapse of mucous membrane is to remove longitudinal portions of it with a clamp and cautery, exactly as for the removal of piles. The scar tissue contracting helps the part to be pulled up, and so narrows the calibre of the bowel. The inflammatory reaction afterwards also, probably, helps the mucous coats to adhere to the muscular coat.

Another method is to take up elliptical or triangular portions of the mucous membrane, and, if necessary, also triangular portions of the anal skin. Then their edges are sutured together with chromicised catgut, and that reduces the calibre of the lower end of the bowel and lessens the opening in the skin. If the condition is too severe for that, it is well dealt with by doing an ordinary Whitehead's operation for piles. That is, separating the mucous membrane all round the anal margin, drawing it down a sufficient distance, partially dividing it, and as it is divided suturing it to the skin edge. Some people have advised that a mixture of carbolic acid, salicylic acid, and bi-borate of soda, with glycerin, should be injected under the mucous membrane, and produce thereby an inflammatory reaction, so as to cause adhesion between the mucous membrane and the other coats of the bowel. I have not personally had any experience of that. I have had only one case of injection, and that was the injection of quinine, of the same formula as used by Mr. Inglis Parsons for prolapse of the uterus: 12 grains sulphate of quinine in

30m dilute sulphuric acid, and 30m distilled water. To do that, the rectum must be cleared out and cleansed as much as possible, and 5 to 6m of that solution injected into the submucous coat at several points by means of a hypodermic syringe. Then the part is replaced and held in position by strapping. Some say that the bowel should be held in position after that injection by means of gauze plugging; up the middle of the plug a tube is passed to allow the flatus to pass. The patient will need to be kept under opium to allow this plug to be retained for several days with any comfort.

### The Coccygeal Operation.

This operation causes plastic exudation between the mucous membrane and the bowel wall, which causes it to adhere. Another method which is fairly often used for causing the mucous membrane to adhere to other coats is to give an anæsthetic, put the patient in the lithotomy position, wash out the bowel as before, pull down the prolapse as far as possible, and with Paquelin's cautery burn about four grooves from one end of the prolapse to the other, burning nearly through the mucous membrane. Then streaks of eucalyptus ointment are laid on the burns, the prolapse returned, and the buttocks held together as before. The patient will have to be kept on his back for three weeks again. Other people advise that exactly similar treatment should be adopted, only using nitric acid; but I do not think that is as satisfactory as the actual cautery, because it is difficult to know how deep you have gone with the nitric acid. A very excellent way of treating procidentia is one which has been practised several times by my colleague here, Mr. Mummery. The patient is given an anæsthetic, put in the left lateral position, a curved incision about two inches long is made between the anus and the coccyx. The incision is deepened until the rectum is reached, and by means of the fingers passed up between the rectum and the sacrum, the rectum is separated from the sacrum for a considerable distance, as far as the attachment of the meso-rectum, and laterally to the attachment of the lateral ligaments of the bowel. If there is any fat adherent to the sacrum, it is scrubbed off by gauze swabs held in forceps, the prolapse is reduced, and then the cavity which is formed is stuffed with sterilised gauze, the object of that gauze being to cause irritation in the parts, so that when the gauze in a few days is gradually removed the rectum will adhere into the hollow of the sacrum, where it ought to be. Several cases have been done in that way, and with remarkably good results. It was always a difficulty to know how long a cure would last after operation. I do not think any case has been done by that method longer than a year, so one can scarcely tell at present what is going to be the after-result. But another good and very effectual method of holding up the procidentia is to do exactly as I have described, and get an assistant to pass a finger into the bowel and force the prolapsed bowel out of the incision which you have made. To give yourself enough room you may have to remove the coccyx. The prolapsed bowel is forced outwards and backwards by an assistant's finger in the rectum, and three or four silkworm gut stitches passed transversely through the muscular coat of the bowel, as high up as possible. These gut stitches are then threaded on a curved needle on a handle, and each end passed upwards in front of the sacrum and passed backwards at the side of the sacrum, until they project on the skin behind. The stitches are then pulled tight, dragging the bowel up to the hollow of the sacrum. They are tied over a pad of gauze to prevent irritation of the skin, and the wound behind the anus is closed, with drainage. The stitches are left in eight to ten days, and the patient kept on his back three weeks. You will see the principle is much the same as in the operation I have just described, where this cavity is stuffed with gauze, which is gradually removed.

### Recurrent Prolapse.

There are some cases where, do what you will, the prolapse returns. Or it may be that you think it is not advisable to try any of these more or less simple methods because the prolapse is very large and the patient's tissues very relaxed, partly because of his age, or because the prolapse comes from too high up the bowel. Then the best thing to do is to open the abdominal cavity on the left side, and draw the bowel up high enough to reduce the whole of the prolapse, and suture a convenient portion of the sigmoid to the abdominal wall. Before the sutures are put in, the peritoneum must be reflected off the abdominal wall, so that the bowel is attached to the transversalis fascia, and not to the peritoneum. There are two reasons for that. If the bowel is attached to the peritoneum only, the adhesions will probably stretch and allow the prolapse to recur. Another reason is that the patients complain of very considerable pain, owing to the dragging on the parietal peritoneum.

### Removal.

There are some cases in which amputation of the prolapsed portion is advisable. Those are cases in which the bowel wall is extensively diseased or strictured, or in which adhesions prevent reduction of the prolapse; and in some cases in which gangrene of the prolapsed portion of the bowel is threatening, where it would not be safe to reduce it. I think the best method of amputating the prolapse is that described as Mickulicz's operation. That is done in this way. The bowel is thoroughly cleared out, the patient put into the lithotomy position, and the prolapse drawn down to its fullest extent. Then an incision is made through the mucous membrane, and the outer tube of the prolapse closed to the anal skin. This incision will be, at first, in front. It is steadily deepened through the whole of the coats of the bowel. By this time the opening is in the peritoneal cavity. I should have said that the patient's pelvis should be elevated on a cushion, so that if there is any bowel fallen down into the pouch formed by the peritoneum it would naturally fall backwards into the abdominal cavity and be out of danger in this operation. A transverse cut is now made through the peritoneum of that inner tube, and the portions of the divided peritoneum of the two tubes are sewn together. This division of the bowel is carried round, so that the peritoneal coats are sutured together. After that, the inner tube of bowel is divided, and the muscular coats and mucous coats of the two tubes are sewn together with thread. A drain tube is passed into the bowel, and the bowel is kept confined for eight days. I always dress the parts with Aristol before putting on the dressings.

Another method which I have had experience of is the injection of paraffin. It is very satisfactory for a time, but the trouble tends to relapse. The operation is easy, and is done by putting the patient in the lithotomy position, bringing the bowel down as far as possible, catching hold of it at three equidistant points with forceps, and injecting a little paraffin midway between each forceps, a volume equal to about three peas. Inject first the higher portion and reduce that; then three more points. The second forceps are applied in a different way: two in front, one behind; the next lot, one in front, two behind. The advantage is that it acts immediately. The cases I have seen done in that way have been all right for some months, but then there is a steady relapse.

The third kind of prolapse which I mentioned was where the bowel does not appear outside the anus, and that merits more description, because it may produce a deal of trouble and may escape recognition. The patients are constipated, they always have a difficulty in emptying the bowel, and when it is emptied they feel that something more needs to come away. They often have dragging pain in the back, passing down the thighs. Owing to the slipping up and down of this prolapsed portion of bowel, the mucous membrane gets inflamed, and may be ulcerated. So that there may be a discharge of more or less mucus or pus; and, if ulceration be present, of blood. Unless careful examination of the bowel is made, the condition is overlooked, and may be confounded with disease of the uterus or ovaries, or other abdominal conditions. The treatment is to open the bowel and suture the sigmoid up.

Another condition which may complicate prolapse in the rectum is a rectal hernia. A pouch of peritoneum may bulge through into the bowel, and in that there may be coils of small intestine. Those coils have, on more than one occasion, prolapsed through a rent in the bowel and appeared in the anus. It is a rather singular fact that whenever this occurs the prolapse is always spontaneously reduced, and only the prolapsed small intestine through the opening appears at the anus. On more than one occasion this intestine has become gangrenous.

